# Sulfate Reduction in Sediments Produces High Levels of Chromophoric Dissolved Organic Matter

**DOI:** 10.1038/s41598-017-09223-z

**Published:** 2017-08-18

**Authors:** Jenna L. Luek, Kaitlyn E. Thompson, Randolph K. Larsen, Andrew Heyes, Michael Gonsior

**Affiliations:** 1 University of Maryland Center for Environmental Science, Chesapeake Biological Laboratory, 146 Williams Street, Solomons, MD 20688 USA; 2 St. Mary’s College of Maryland, Department of Chemistry, 47645 College Drive, St. Mary’s City, MD 20686 USA

## Abstract

Sulfate reduction plays an important role in altering dissolved organic matter (DOM) in estuarine and coastal sediments, although its role in the production of optically active chromophoric DOM (CDOM) and a subset of fluorescent DOM (FDOM) has not been previously investigated in detail. Freshwater sediment slurries were incubated anaerobically with added sulfate and acetate to promote sulfate-reducing bacteria. Ultraviolet visible (UV-Vis) absorbance and 3-dimensional excitation emission matrix (EEM) fluorescence spectra were measured over a five weeks anaerobic dark incubation period. Parallel Factor Analysis (PARAFAC) of FDOM determined components that increased significantly during dark and anaerobic incubation matching three components previously considered of terrestrially-derived or humic-like origin published in the OpenFluor database. The observed FDOM increase was strongly correlated (R^2^ = 0.96) with the reduction of sulfate. These results show a direct experimental link between sulfate reduction and FDOM production, which impacts our understanding of coastal FDOM sources and early sediment diagenesis. As 3D fluorescence techniques are commonly applied to diverse systems, these results provide increasing support that FDOM can have many diverse sources not consistently captured by common classifications such as “humic-like” fluorescence.

## Introduction

Aquatic sediments process and store large quantities of organic matter with preservation determined by the balance of loss and accumulation rates^1, 2^. DOM is extremely complex and individual molecules are degraded at highly variable rates^3–5^. Degradation of DOM in terrestrial and marine ecosystems can be described along a continuum where simple molecules such as acetate, carbohydrates and proteins are preferentially utilized followed by other more stable molecules such as lignin and melanoidins, while some DOM remains recalcitrant^3, 6^. DOM in aquatic sediments has a wide range of reactivities and may be modified both biotically and abiotically, resulting in up to 99.5% remineralization^2, 6–9^. DOM that is not subjected to fast degradation or remineralization may be flocculated, preserved and buried as sedimentation continues^2, 3, 10^. Anoxia, physical protection, and specific chemical reactions such as condensation and aggregation play key roles in organic matter burial efficiency in coastal sediments^1^.

Sediment and porewater DOM can be utilized by terminal respiratory processes such as iron and manganese reduction, sulfur reduction, or methanogenesis under anaerobic conditions^11, 12^. Organic carbon mineralization in anaerobic sediments by dissimilatory sulfate reducing bacteria (SRB) is thought to be the main terminal respiratory process in continental margin sediments^13^, and the rate of sulfate reduction depends on temperature and the quality and quantity of labile DOM^5^. SRB have the ability to reduce dissolved sulfate into inorganic sulfur species such as hydrogen sulfide and polysulfides and utilize labile DOM in pore waters^14^. Because of the dependence of SRB on the supply of sulfate, bioavailable carbon, and nutrients, the incorporation of sulfur in organic matter is linked to processes in near surface sediments with high rates of bacterial sulfur reduction and the concomitant formation of iron sulfides^15–17^. However, SRBs are also in competition with other microbes for energy resources, such as with methanogens, and often co-exist in the environment. As such, sulfate availability often controls the transition from sulfate reduction to fermentation in anoxic estuarine sediments^18^.

Fluorescent dissolved organic matter (FDOM), the component of DOM that can absorb and fluoresce, has been shown to increase with depth in sediment pore waters^19, 20^. Excitation-emission matrix (EEM) fluorescence paired with parallel factor analysis (PARAFAC) provides a powerful tool used to quantitatively describe FDOM^21, 22^. Despite the extremely high diversity of organic compounds found in the environment, a handful of dominant fluorescence regions are consistently observed in EEM-PARAFAC components in both natural and engineered aquatic environments^23, 24^. The fluorescence peak frequently described as “humic-like” fluorescence is relatively stable in the environment^24, 25^, but may be photolabile over longer time periods^26, 27^. Additionally, this “humic-like” fluorescence shows different biolability after photodegradation^28^. Humic substances are thought to be preferentially preserved in anoxic sediments, as are sulfur containing humic substances that show humic-like fluorescence^9, 16, 19, 20^.

Field studies and incubation experiments with open ocean waters have indicated a link between increasing microbial metabolism and increasing FDOM, such as in water column oxygen minimum zones^29–32^. The addition of labile carbon (e.g., glucose, acetate) can alter the FDOM increase during incubations^30, 32^, and may indicate that microbial metabolism of less-labile carbon sources results in high levels of “humic-like” fluorescence^32, 33^. Variable FDOM changes have been observed in laboratory studies investigating FDOM during the growth and/or decay of specific phytoplankton^28, 34–36^ and seagrasses^37, 38^. In coastal ocean sediments, a negative correlation between FDOM intensity and sulfate concentrations has also been observed^39, 40^.

In this study, we investigated the production pathways of FDOM utilizing labile acetate and hypothesized an increase in FDOM through optimal growth conditions for sulfate-reducing bacteria in coastal sediments. Using PARAFAC modeling of EEM fluorescence, we evaluated the production of FDOM directly related to sulfate-reducing bacteria and fermenting bacteria in anaerobic sediment pore waters.

## Results

### Sulfate Depletion

**Figure 1:**
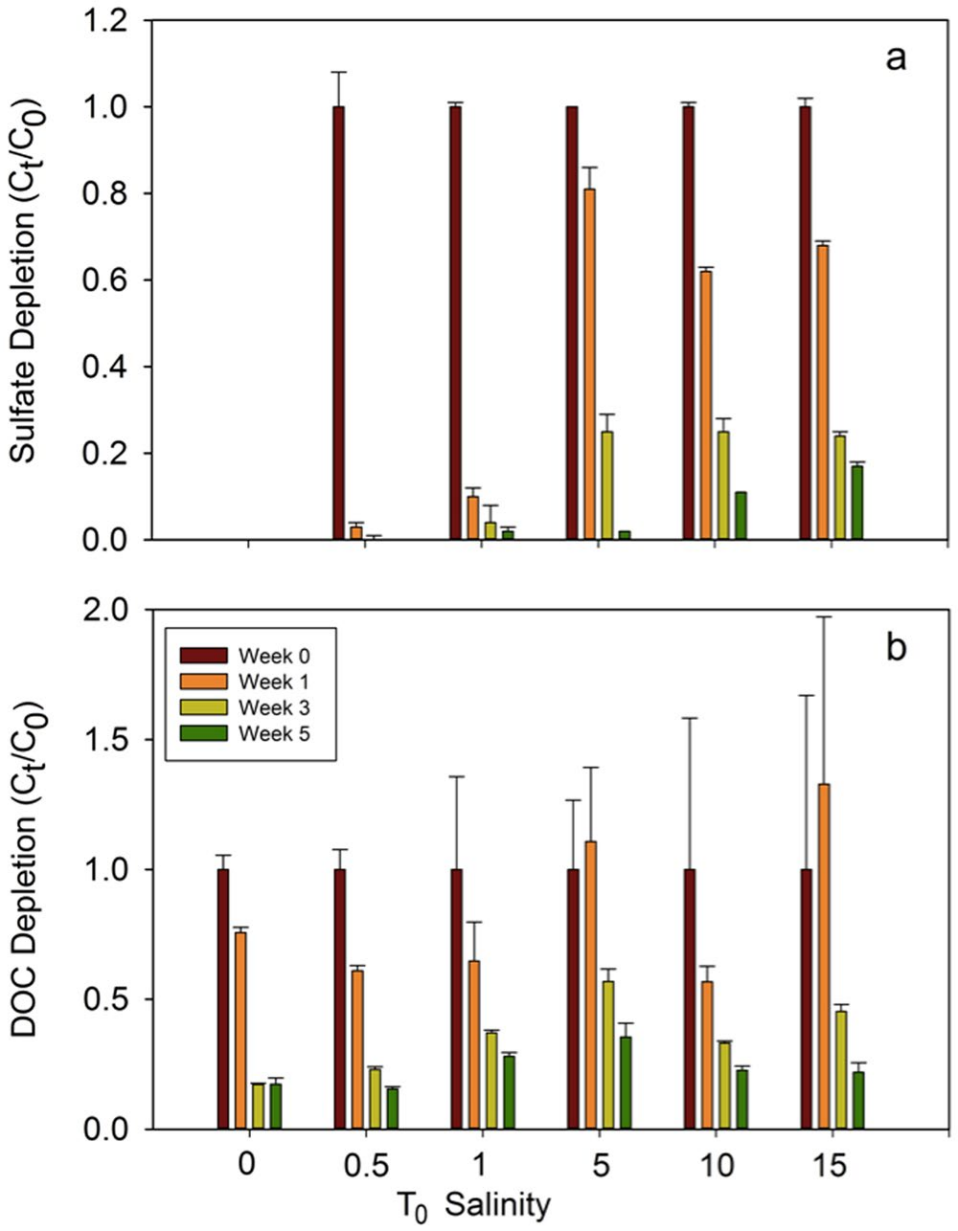
Changes in sulfate (**a**) and DOC (**b**) concentrations relative to initial concentration over a 5 week dark and anaerobic incubation period. Initial sulfate concentrations were equivalent to salinity* 0–15 and DOC concentrations are a combination of background DOC and acetate added in stoichiometric proportions to sulfate. Error bars represent standard error.

Dissolved organic carbon (DOC) and sulfate decreased over the five-week incubation for all treatments (Fig. 1, Supplementary Figs S2, S3). In the control containing no additional sulfate or acetate, a 78% reduction in DOC was observed over five weeks. DOC removal was likely due to the anaerobic remineralization of labile carbon present in the sediment porewaters, although it cannot be ruled out that some may have been removed from the dissolved phase by sorption to particles. Variability in initial DOC and sulfate concentrations indicated that samples collected on Day 0 were not well mixed prior to the first sampling. The rate of sulfate depletion was the highest within the first week, and was completely removed in one week in the sulfate equivalent salinity of 0.5 and 1 treatments. At salinity* 5, 10, and 15 treatment levels, the rates of depletion were indicative of an active growing SRB community, although cells were not counted. A clear first order decay rate was observed for the removal of sulfate in the salinity* 10 and 15 treatments (k = 0.0624 d^−1^, k = 0.0535 d^−1^); similar decay rates were calculated for DOC for these two treatments (k = 0.0579 d^−1^, k = 0.0544 d^−1^). In repeated experiments with added Suwannee River Natural Organic Matter (SRNOM) IHSS standard and iron addition, DOC removal rates remained consistent (Supplementary Table S2). Sulfate removal rates were the same when SRNOM was added, but increased when iron was added (k = 0.1277 d^−1^, k = 0.0895 d^−1^ at the salinity* 10 and 15 treatments, respectively). Dissolved sulfide measured during SRNOM and SRNOM/iron addition experiments revealed sulfide was present at low levels. Sulfide concentrations were near the detection limit (0.3 μM HS^−^) with the highest sulfide concentrations measured on day 21 (3.7 μM). This confirms that sulfate was indeed being reduced to sulfide, however the produced sulfide was likely rapidly lost to inorganic precipitates (e.g., FeS), evasion and possibly conversion to organic sulfur species^15, 16^.

### Increase in fluorescence

**Figure 2:**
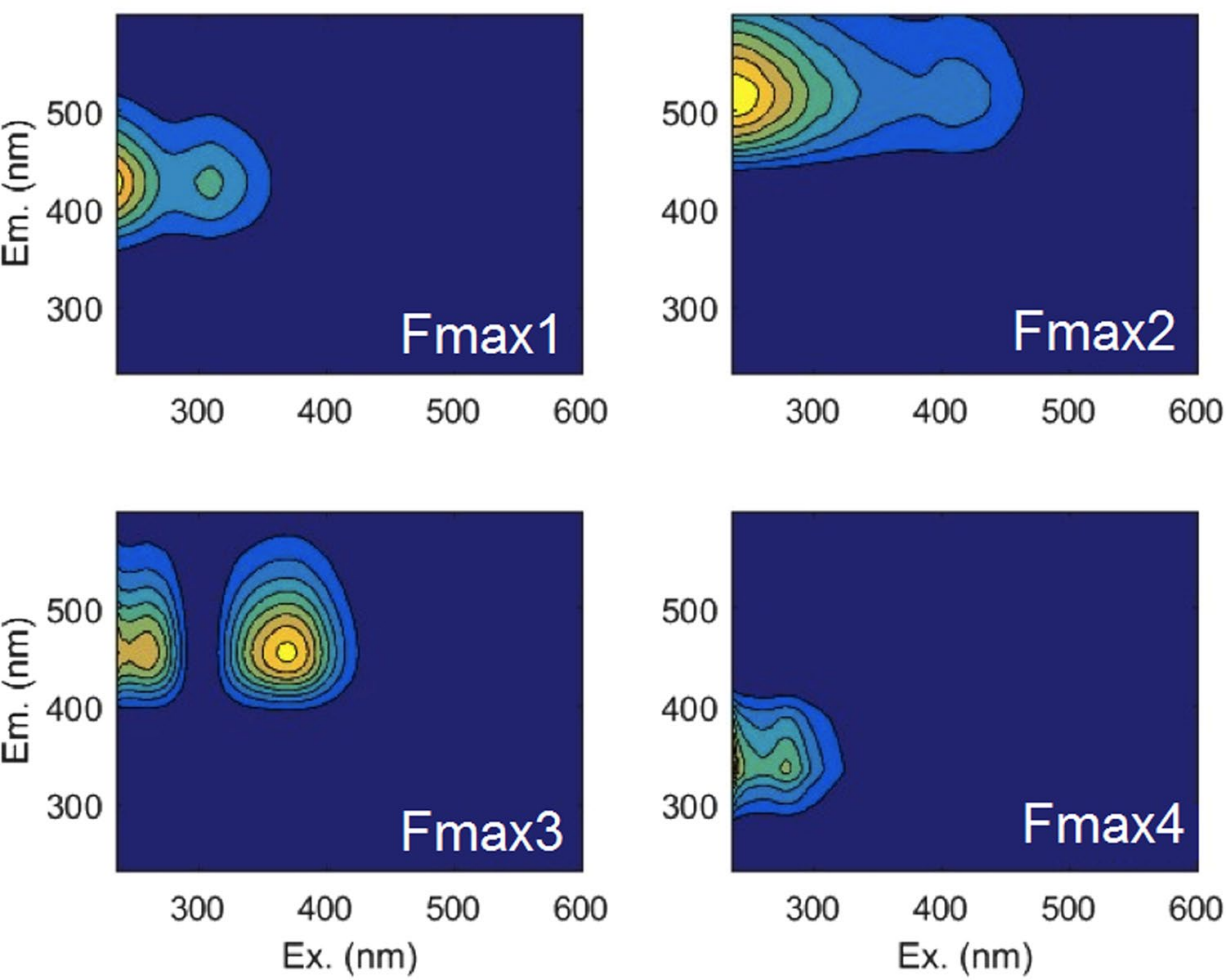
EEM**-**PARAFAC statistical components Fmax1–4 of fluorescence observed in all samples.

A clear increase in CDOM was observed over the duration of the experiment, with darker coloration in the higher sulfate treatments. Initial fluorescence was low and the four-component EEM-PARAFAC model (Fig. 2) revealed increases in three of the four components (Fmax1, Fmax2, Fmax3) in the salinity* 5, 10, and 15 treatments. No change in component 4 (Fmax4) was observed over time. The low salinity* treatments (0.5, 1) had no clear increase in fluorescence, similar to the no sulfate added control. The largest increases were observed in Fmax1 and Fmax2, where the initial fluorescence increased as much as five fold over the initial fluorescence in the 15 ppt treatment. UV-Vis absorbance increased over the incubation, and the absorbance at 254 nm was linearly related to Fmax1, Fmax2, and Fmax3. The relationship between fluorescence and absorbance indicated that the CDOM material produced contained humic-like fluorophores.

The addition of SRNOM resulted in an increase in initial fluorescence for components Fmax1, Fmax2, and Fmax3, but then closely traced the original experimental results for the duration of the experiment. Interestingly, a clear increase in fluorescence was not seen on day 1 when iron was also added. Dissolved iron may have resulted in a quenching of fluorescence^41^. Fmax4 had consistently low intensities but varied across experiments, with the highest fluorescence observed when iron and SRNOM were added and second highest when only SRNOM was added.

Components Fmax1, Fmax2, and Fmax3 are among the most frequently identified fluorescence patterns observed in natural water samples^24^; Fmax2 and Fmax3 are generally considered more stable than Fmax1, although all are traditionally considered “humic-like” fluorescence^24^. Fmax4 is characteristic of a “protein-like” fluorescent peak, often associated with the two fluorescent amino acids tyrosine and tryptophan in porewater samples^23^ and heterotrophic bacteria^42^, although other sources for this fluorescence signal cannot be ruled out. The PARAFAC model was compared to published PARAFAC models from the database OpenFluor^43^ and a large number of models from aquatic systems matched individual components within the minimum similarity score of 0.95 (Fmax1-25, Fmax2- 17, Fmax3- 2, and Fmax4- 9 models) (Supplementary Figs S4, S5). An analysis of Arctic river FDOM^44^ matched all four components, and their components matching Fmax1, Fmax2, and Fmax3 were correlated with lignin phenol content and mosses and peat^44^. Although the fluorescence components described in our PARAFAC model are ubiquitous in DOM, they have never been associated specifically with SRB processes and SRB metabolites in sediment porewaters prior to this research.

## Discussion

**Figure 3:**
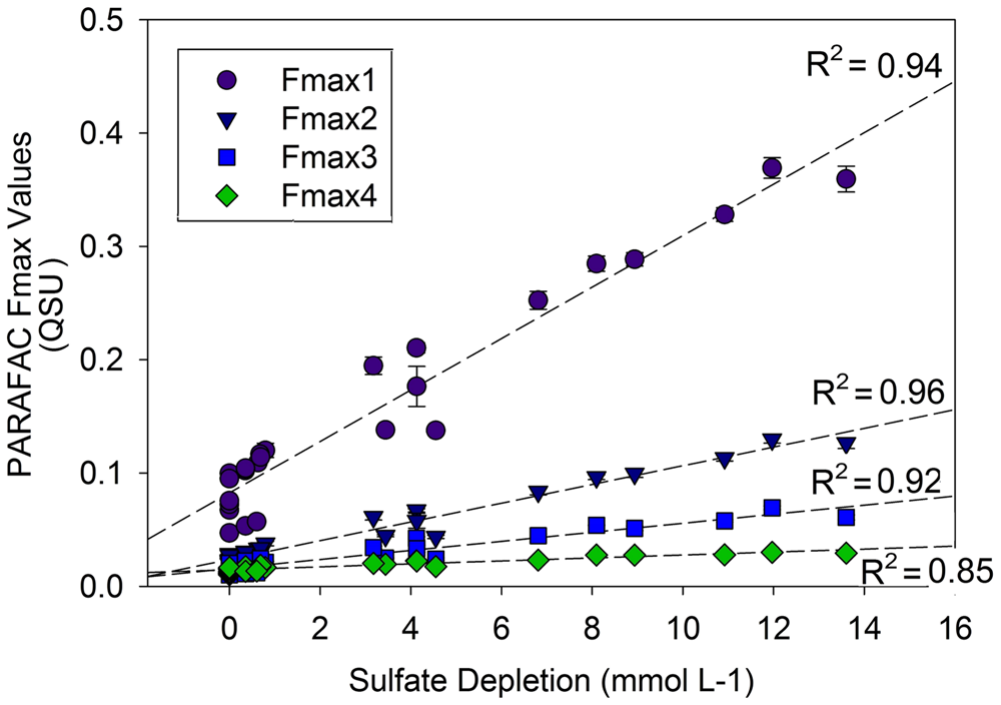
Sulfate depletion versus the intensity of individual PARAFAC components (Fmax1, Fmax2, Fmax3, Fmax4) in quinine sulfate equivalents (QSU) within each sample. A linear regression for each component is shown, error bars represent standard error of the mean. Standard error of slope: C1: 0.023 ± 0.001, C2: 0.0083 ± 0.00034, C3: 0.004 ± 0.00023, C4: 0.0013 ± 0.0001.

A strong correlation was observed between sulfate depletion and EEM-PARAFAC components (Fig. 3, Supplementary Figs S6, S7). Two prior studies have shown observational evidence relating an increase in fluorescence to decreasing sulfate concentrations at depth in coastal ocean sediments^39, 40^. The source of FDOM formed during sulfate reduction may be related to a number of different reactions: 1) the conversion of particulate organic matter (POM) to DOM^1, 7, 45^ 2) formation of thiophenes under reducing conditions^46, 47^ 3) direct or secondary metabolite release from the SRB community 4) a microbial degradation product directly linked to SRB source material. A prior study identifying “humic-like” FDOM production using only a glucose carbon source in artificial seawater and a microbial inoculate support this assertion that FDOM production may be associated with compounds released from the microbial community^30^. A possible metabolite arising from the degradation of porphyrins such as siroheme, which is a highly carboxylated, water-soluble tetrapyrrole and intense chromophore^48^ might explain the observed fluorescence, but siroheme would need to be severely degraded to be able to show absorbance and fluorescence in the “humic-like” range. The result for such a degradation would likely yield pyrrolic acid-type compounds that potentially would show the observed humic-like signals (Fmax1–3). The pyrrolic sub-structure may be able to produce fluorescence with a large Stokes shift, similar to that of polyphenols in “humic-like” FDOM. Prior work has also suggested that the degradation of amino-acid fluorophores may also lead to the production of “humic-like” fluorescence^49^.

**Figure 4:**
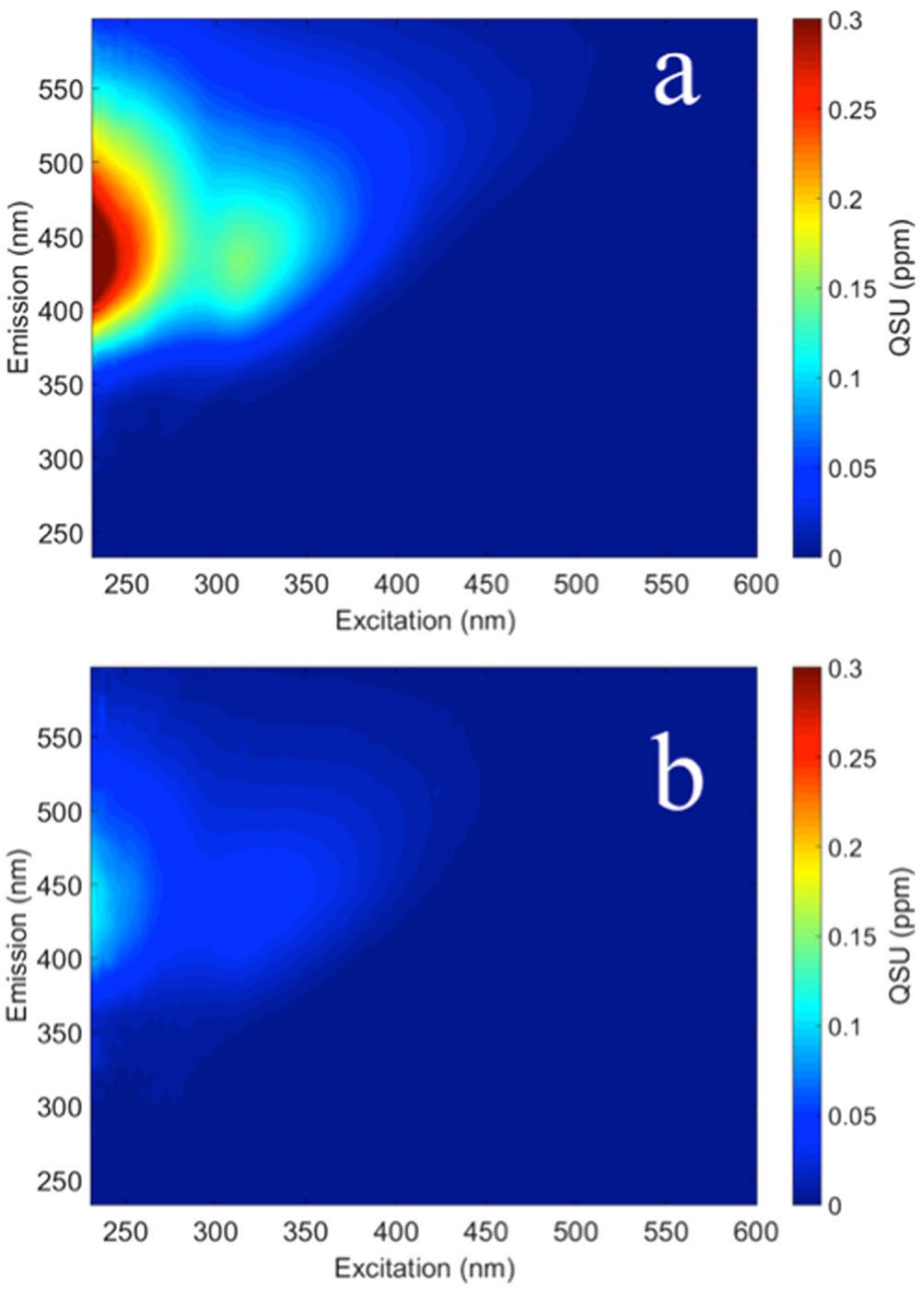
Increase in fluorescence over a 5 week dark and anaerobic incubation of freshwater sediments with sulfate/acetate treatment (**a**) and acetate only treatment (fermentation) (**b**).

Under anaerobic conditions, a number of other terminal electron acceptors play a role in the reworking of DOM. To test if these non-sulfate pathways resulted in the production of FDOM, two controls were tested. Controls containing no sulfate or acetate did not result in an increase in fluorescence as discussed previously (Fig. 3). Fermentation was compared to SRB-related degradation by adding only labile carbon as acetate, and did result in the production of FDOM. However, FDOM was produced to a much lesser degree than the sulfate treatment containing the same quantity of acetate (Fig. 4), although DOC was removed at a faster rate during the fermentation experiment (k = 0.084 d^−1^ [fermentation] vs k = 0.057 d^−1^ [sulfate]). FDOM production during fermentation occurred in the same four dominant regions characterized by the PARAFAC model (Supplementary Fig. S8), but had a different overall absorbance spectra (Supplementary Fig. S9). Fermentation by *Saccharomyces cerevisiae* has been shown to produce FDOM that closely resembled the spectra of tryptophan, nicotinamide adenine dinucleotide (NADH), and riboflavin^50^. The Fmax3 fluorescent component closely resembled the fluorescent peak of *S. cerevisiae* component presumed to be related to NADH by the authors^50^. Although fermentation may play a role in FDOM production, SRB activity was much more strongly correlated with FDOM.

This study provides direct experimental evidence of the production of optically active FDOM material during sulfate reduction and fermentation. Prior field studies have identified an inverse correlation between sulfate concentrations and FDOM intensity^39, 40^. In this study, we simulated the movement of freshwater sediment organic matter through estuarine sediments by altering sulfate availability under anaerobic conditions. Samples were allowed 1–2 weeks to oxygenate following the anaerobic experiment, and fluorescence decreased across all experiments and treatments. However, final fluorescence remained elevated up to five-fold above initial conditions in the Fmax1 component. These results indicate that when anoxic sediments get resuspended or oxygenated from overlying waters, a portion of the newly created FDOM is also likely to be relatively stable under these new conditions.

FDOM distributions have played a key role in advancing our understanding of biogeochemical processing of organic matter in aquatic systems^51–53^. This study indicates that sulfate reduction plays a clear role in the formation of “humic-like”^23^ fluorescence although these compounds are not known to be of humic origin. It is becoming increasingly apparent that the spectral regions originally designated as “humic-like” likely have non-humic sources^29, 54^. Indeed, coastal phytoplankton have also recently been described as potential sources of the FDOM signature previously described as “humic-like” and terrestrially derived^28, 34–36^, as have bacterial degradation of simple carbon sources^30^, fermenting bacteria^50^, and simple cellular compounds including NADH^50^. The results of this study warrant further research into the role sulfate reducing bacteria play in the production of FDOM, including varying carbon sources^30, 32^, identifying fluorescent microbial products and metabolites, and investigating this relationship in natural systems^39, 40^.

## Methods

### Experimental Design

Anaerobic sediment slurries were used to investigate the relationship between sulfate addition and FDOM production under anaerobic conditions in sediment pore waters. Freshwater sediments were collected upstream of the Conowingo Dam on the Susquehanna River and mixed with varying concentrations of sulfate and acetate to provide optimal conditions for SRB growth. The experiment was carried out under anaerobic conditions using a Coy® Glove Bag, and experimental vials were sampled following the addition of amendments (day 1) and on days 7, 21, and 35. This experiment was repeated using 1) a standard natural organic material (Suwannee River Natural Organic Matter-SRNOM, IHSS standard) amendment and 2) SRNOM and iron (III) oxide amendments (Supplementary Table S1).

All samples were transferred and incubated in clean 250 mL Schott glass bottles (baked 500 °C 5 h) and kept at 20 °C in the dark. Each sample contained a slurry of 150 g freshwater anaerobic Susquehanna River sediment and 200 mL of degassed Milli-Q water. Milli-Q water was degassed by boiling, purging with nitrogen gas for 1 hr prior, and cooling, then combined with sediment samples and rested for one week under anaerobic conditions prior to adding amendments.

Sediment slurries were amended with sodium sulfate in six triplicate vials corresponding to a sulfate salinity equivalence gradient of 0, 0.5, 1, 5, 10 and 15 ppt (77 μM, 155 μM, 786 μM, 1.58 mM, and 2.37 mM), herein referred to simply as salinity*. Sodium acetate was added in stoichiometric proportions (1.7:1 sodium sulfate to sodium acetate) to promote sulfate reduction under anaerobic conditions. Sediment slurries containing high acetate (194 mg) and no sulfate were mixed to investigate the role of fermentation alone on FDOM production.

On days 1, 7, 21, and 35, 20 mL water samples were vacuum filtered through 0.7 μm GF/F filters. Following the 5-week anaerobic incubation, sediment slurries were exposed to ambient oxic conditions for an additional 1 or 2 weeks and subsequently sampled in the same manner. Filtered samples were analyzed for sulfate, sulfide, DOC, absorbance and EEM fluorescence. Sulfide analyses were run within 24 hours of collection, absorbance and fluorescence samples were run within 5 days. Sulfate samples were immediately acidified to a pH of 2 with hydrochloric acid. DOC and acidified sulfate samples were stored in amber glass vials and stored at −18 °C until analysis was performed.

### Analytical Approaches

Sulfide concentrations were measured with a sulfide and reference electrode described previously^55^. A sulfide anti-oxidant buffer (SAOB) containing sodium hydroxide, sodium EDTA, and ascorbic acid in deoxygenated water and was added to each samples in a volumetric ratio of 1:1 prior to analysis. Sulfate analysis was performed on thawed, diluted (1:10) using a Dionex Ion Chromatograph^56^. Thawed samples were analyzed for dissolved organic carbon (DOC) as non-purgeable organic carbon with an automatic total organic carbon analyzer (Shimadzu TOC-V) by oxidation at 680 °C with a platinum catalyst column. Samples were internally acidified with HCl to pH 2, sparged for 2.5 min to remove inorganic carbon, and quantified using potassium hydrogen phthalate standards.

UV-Vis absorbance and EEM fluorescence measurements were obtained using a temperature-controlled Jobin Yvon Horiba Aqualog fluorescence spectrometer. Inner filter correction was then carried out using the simultaneously measured UV-Vis absorbance and the algorithm available within the Horiba Aqualog software. Samples were diluted with Milli-Q water (volumetric ratio 1:5) to not exceed 0.4 raw absorbance at 300 nm and to be able to perform adequate inner filtering correction. The fixed emission was recorded using the Aqualog CCD detector over the range from 200–600 nm at excitation wavelengths ranging from 230–600 nm at 3 nm intervals. Fluorescent intensities were Rayleigh scatter corrected and normalized to a STARNA quinine sulfate fluorescence standard of 1 ppm concentration. Statistical PARAFAC analysis^22, 57^ was applied to the EEM dataset by using the drEEM toolbox, developed in Matlab and utilizing the N-way toolbox^21^. A 4-component model was most suitable to explain the fluorescence data, split-half validation was performed and the 4-component model was validated (Supplementary Fig. S1).

The datasets generated during and/or analyzed during the current study are available from the corresponding author on reasonable request.

## Electronic supplementary material

Supplemental File
