# Author Correction: Sulfate Reduction in Sediments Produces High Levels of Chromophoric Dissolved Organic Matter

**DOI:** 10.1038/s41598-018-25424-6

**Published:** 2018-05-01

**Authors:** Jenna L. Luek, Kaitlyn E. Thompson, Randolph K. Larsen, Andrew Heyes, Michael Gonsior

**Affiliations:** 1 University of Maryland Center for Environmental Science, Chesapeake Biological Laboratory, 146 Williams Street, Solomons, MD 20688 USA; 2 St. Mary’s College of Maryland, Department of Chemistry, 47645 College Drive, St. Mary’s City, MD 20686 USA

Correction to: *Scientific Reports* 10.1038/s41598-017-09223-z, published online 18 August 2017

This Article contains typographical errors in the Acknowledgements section.

“This is contribution (XXXX, to be filled in after acceptance of manuscript) of the University of Maryland Center for Environmental Science, Chesapeake Biological Laboratory.”

should read:

“This is contribution 5394 of the University of Maryland Center for Environmental Science, Chesapeake Biological Laboratory.”

