# Supplementary material for: Sulfate Reduction in Sediments Produces High Levels of Chromophoric Dissolved Organic Matter
Source: Sci Rep. 2017 Aug 18;7:8829. doi: 10.1038/s41598-017-09223-z (PMC5562794; doi:10.1038/s41598-017-09223-z)
Supplement: Supplementary file 1 — Supplemental File [file 41598_2017_9223_MOESM1_ESM.pdf]

Supplemental Material

Sulfate Reduction in Sediments Produces High Levels of Chromophoric Dissolved Organic Matter

Jenna L. Luek[1], Kaitlyn E. Thompson[1,2], Randolph K. Larsen III[2], Andrew Heyes[1] Michael Gonsior[1]

1. University of Maryland Center for Environmental Science, Chesapeake Biological Laboratory, Solomons, MD, USA
2. St. Mary's College of Maryland, St. Mary's City, MD, USA

Contents

**Table S1.** Experimental Setup	3
**Figure S1**. Split-half validation of four component PARAFAC model	4
**Figure S2.** Sulfate and DOC changes (Expt. 2)	5
**Figure S3**. Sulfate and DOC changes (Expt. 3)	6
**Figure S4**. OpenFluor comparison Fmax1 & Fmax2	7
**Figure S5**. OpenFluor comparison Fmax3 & Fmax4	8
**Table S2**. First order rate constants for sulfate and DOC	9
**Figure S6**. Sulfate versus PARAFAC components (Expt. 2)	10
**Figure S7**. Sulfate versus PARAFAC components (Expt. 3)	11
**Figure S8.** Fluorescence during acetate only addition	12
**Figure S9**. Absorbance coefficient comparison	13

Table S1. Additions of sodium sulfate, sodium acetate, and/or Suwannee River NOM or iron oxide to sediment slurry samples in all experiments. All slurries contained 150 g sediment and 200 mL oxygen-free MilliQ water. **Note: Salinity equivalents are the salinity corresponding to the specific concentrations of sulfate added.*

Experiment	Salinity Equivalents* (ppt)	Sodium Sulfate (mg/L)	Sodium Acetate (mg/L)	Suwannee River NOM (mg)	Iron (III) Oxide (mg)
1	0	0	0	n/a	n/a
	0.5	11	6.5		
	1	22	13		
	5	121	65		
	10	224	129		
	15	336	194		
2	0	0	0	10	n/a
	0.5	11	6.5	10	
	1	22	13	10	
	5	121	65	10	
	10	224	129	10	
	15	336	194	10	
	0 (acetate only)	-	194	-	
3	0	0	0	10	5
	0.5	11	6.5	10	5
	1	22	13	10	5
	5	121	65	10	5
	10	224	129	10	5
	15	336	194	10	5
	0 (acetate only)	-	194	-	5

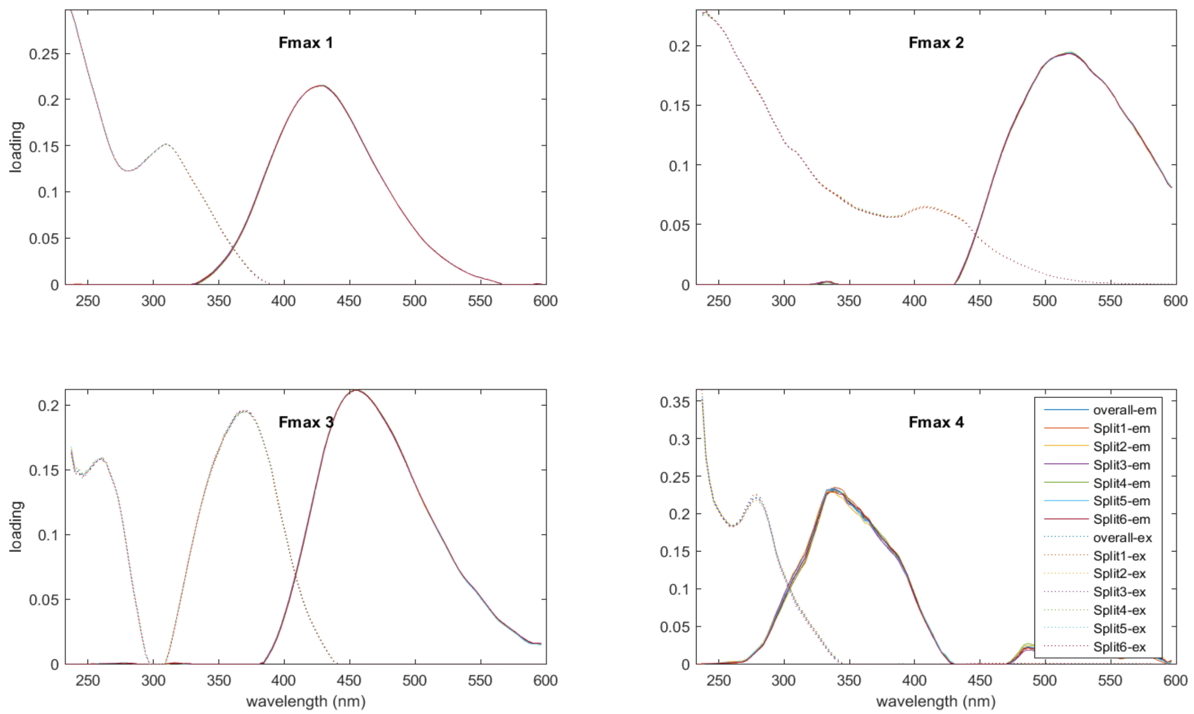

Fig. S1. Split-half validation of the four component EEM-PARAFAC model.

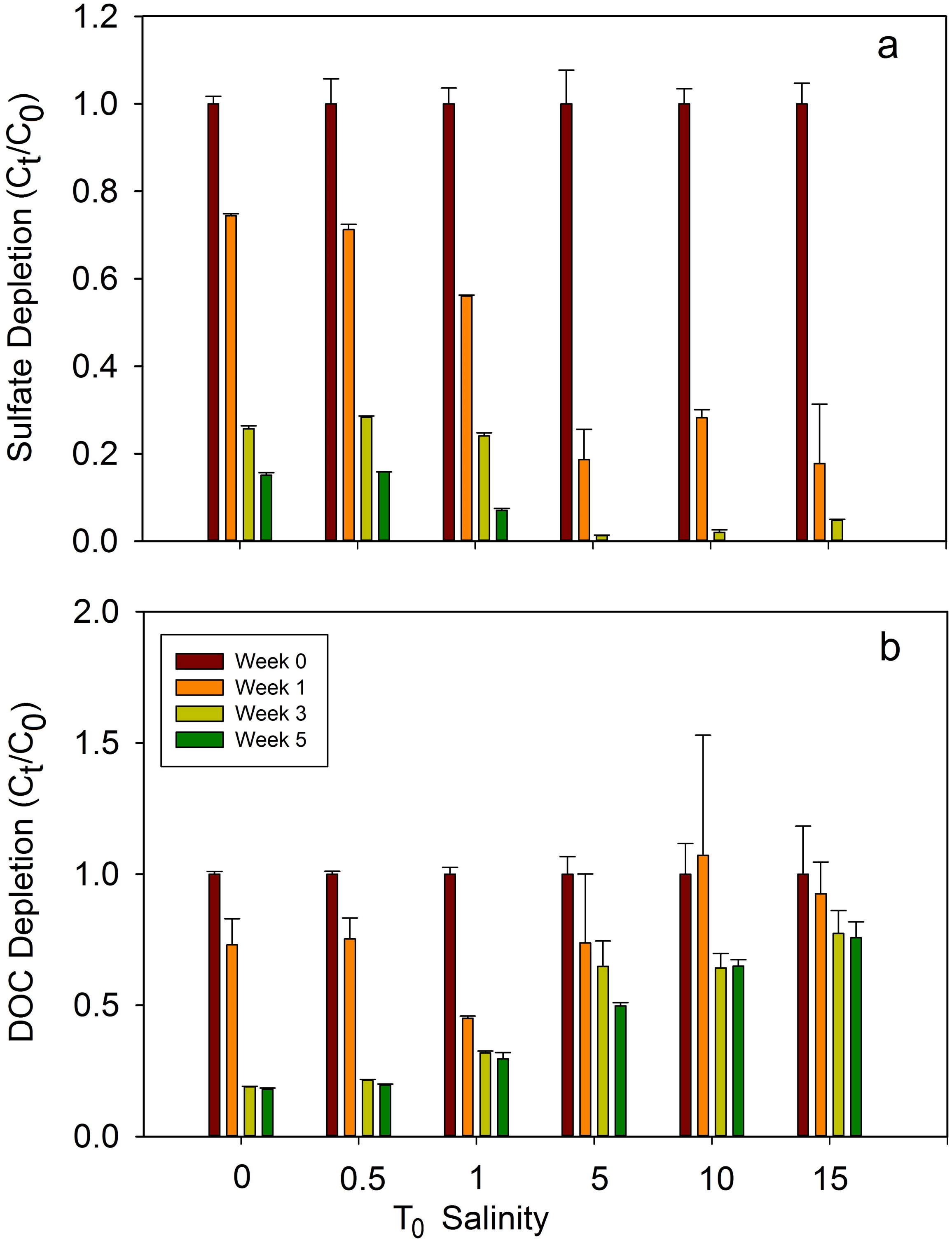

Fig. S2. Changes in sulfate (a) and DOC (b) concentrations relative to initial concentration during a 5 week dark and anaerobic incubation experiment with the addition of SRNOM (Experiment 2). Initial sulfate concentrations were equivalent to salinity of 0-15 and DOC concentrations are a combination of background DOC and acetate added in stoichiometric proportions to sulfate. Error bars represent standard error.

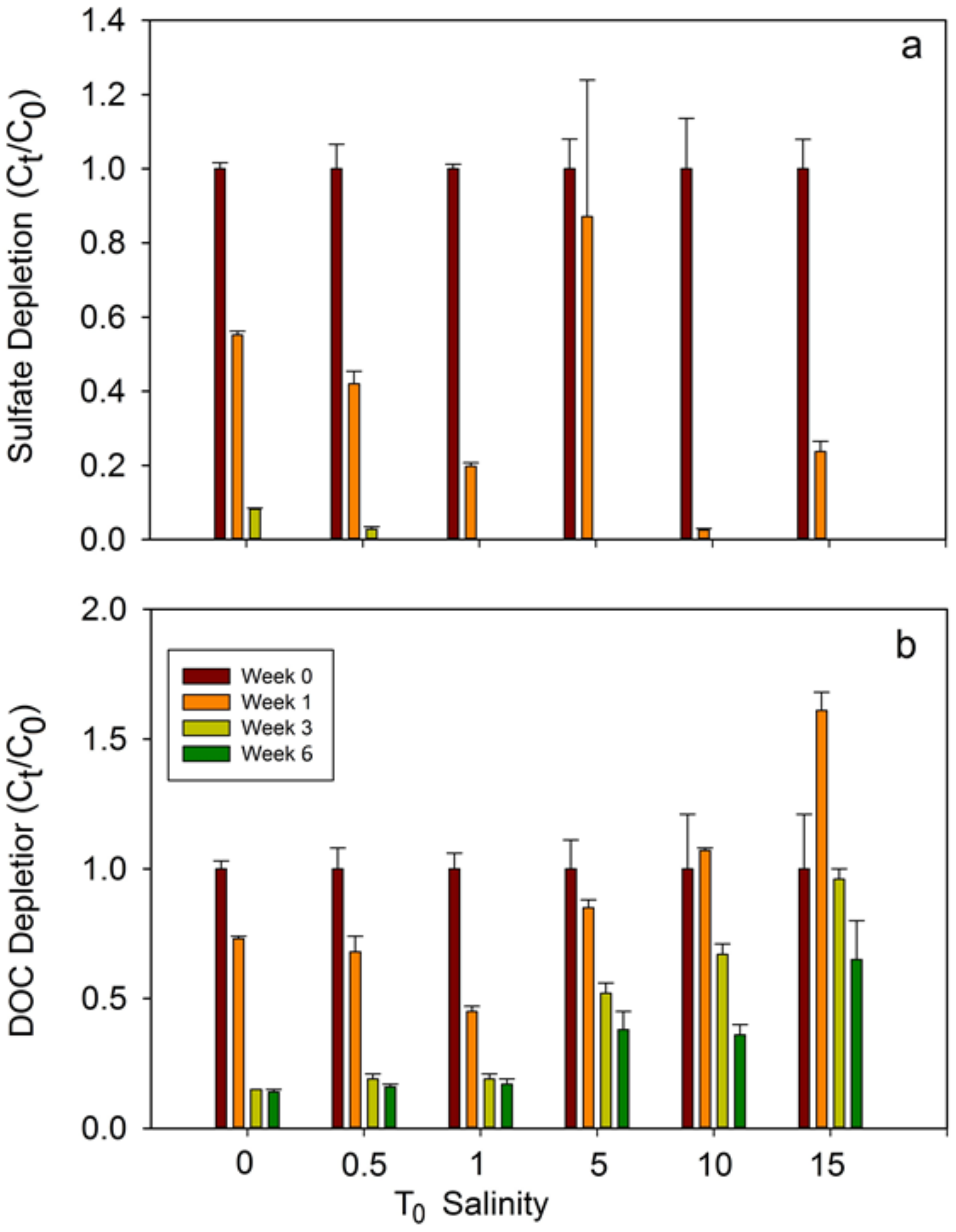

Fig. S3. Changes in sulfate (a) and DOC (b) concentrations relative to initial concentration during a 6 week dark and anaerobic incubation experiment with the addition of SRNOM and Fe(III) oxide (Experiment 3). Initial sulfate concentrations were equivalent to salinity 0-15 and DOC concentrations are a combination of background DOC and acetate added in stoichiometric proportions to sulfate. Error bars represent standard error.

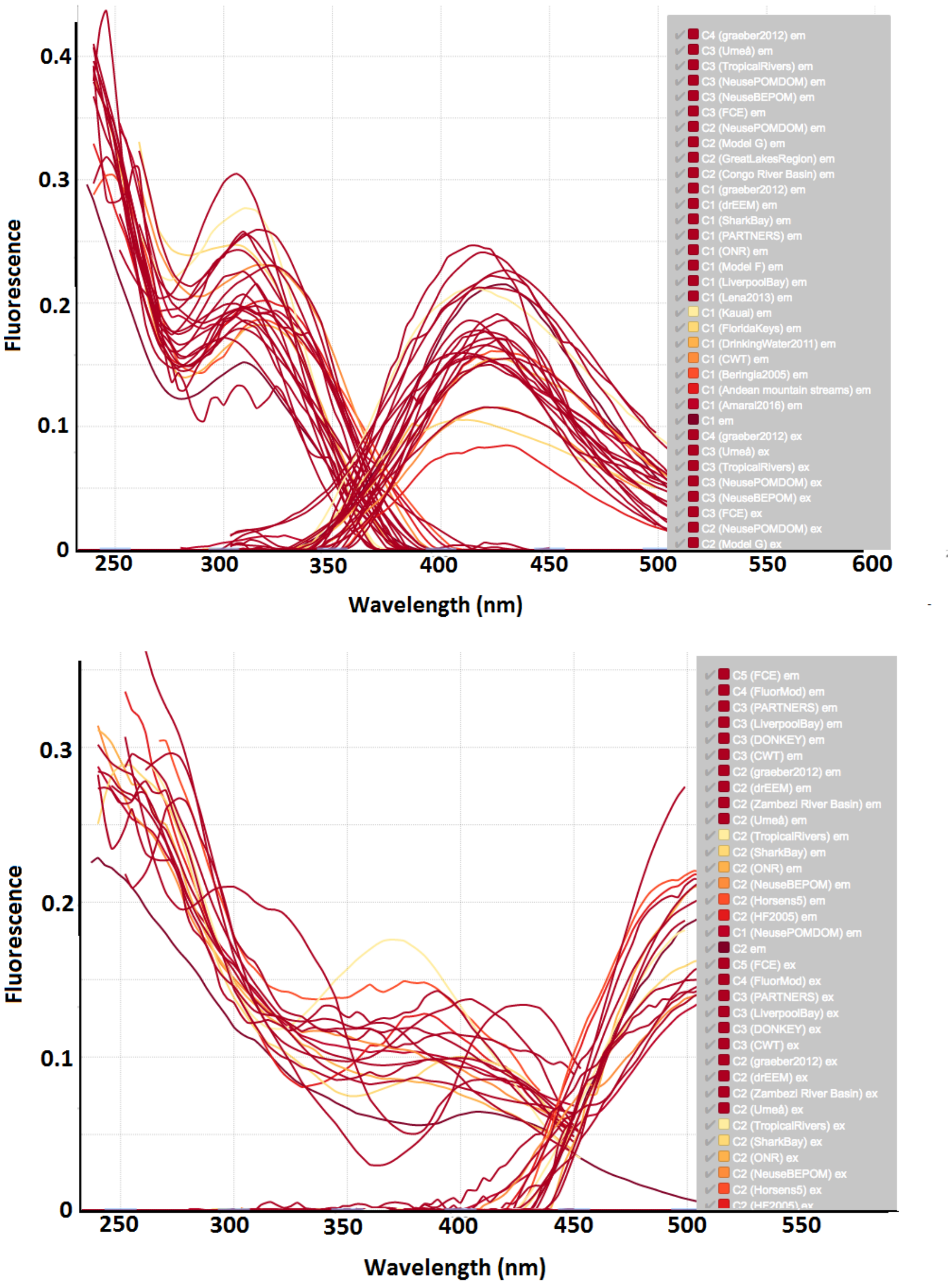

Figure S4. Comparison of modeled EEM-PARAFAC component Fmax1 (top, C1 em) and Fmax2 (bottom, C2 em) to published datasets using the OpenFluor database.

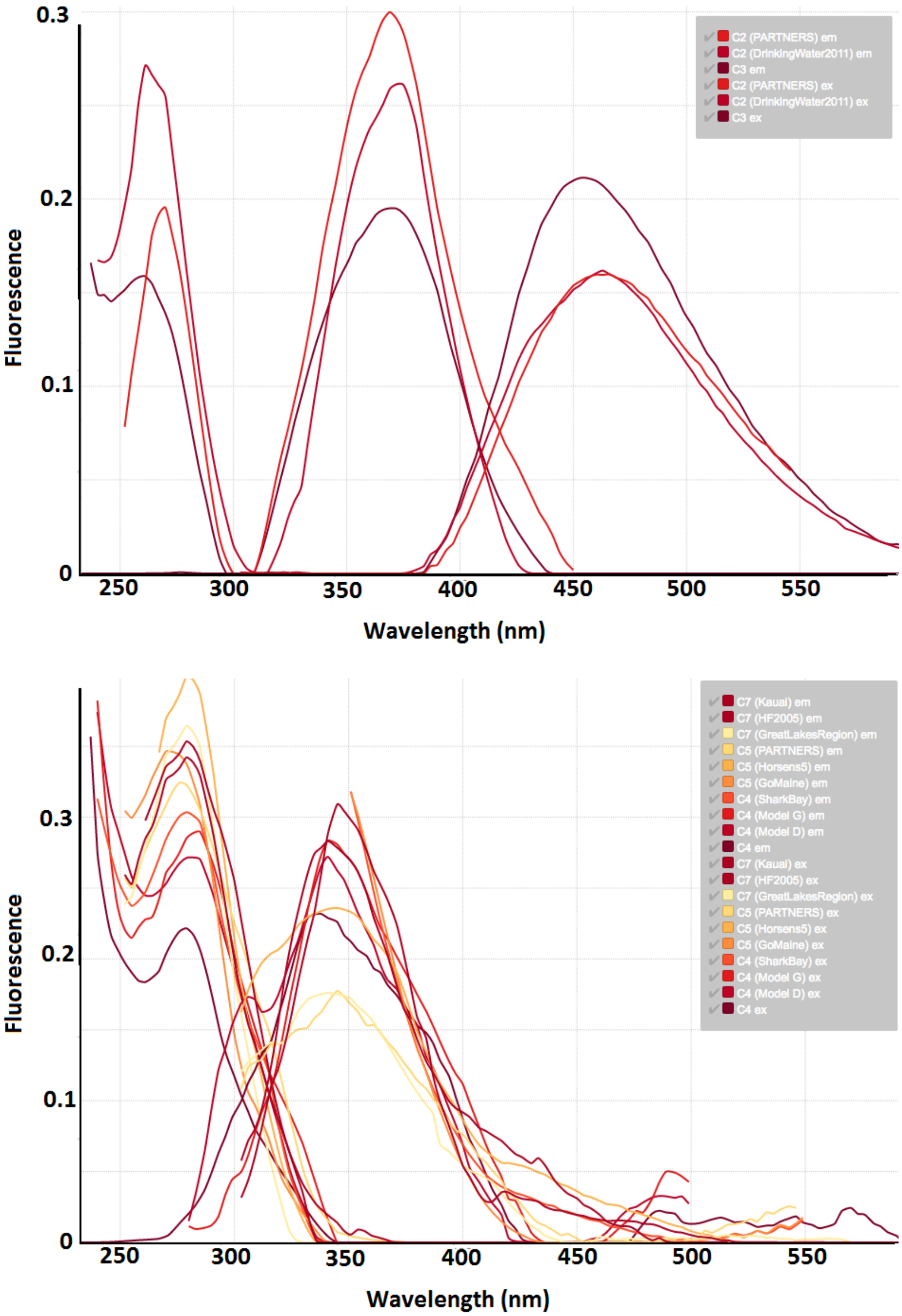

Figure S5. Comparison of modeled PARAFAC component Fmax3 (top, C3 em) and Fmax4 (bottom, C4 em) to published datasets using the OpenFluor database.

Table S2. First order rate constants (k, d^{-1}) for sulfate and DOC during individual experiments for high salinity treatments.

	k Sulfate (d^{-1})	R^2 first order decay	k DOC (d^{-1})	R^2 first order decay
Expt. 1				
5	0.1117	0.93	0.0395	0.78
10	0.0624	0.99	0.0544	0.96
15	0.0535	0.96	0.0579	0.86
15 (fermentation, no sulfate)	NA	NA	0.0845	0.94
Expt. 2				
5	0.074	0.99	0.031	0.75
10	0.0541	0.99	0.0512	0.89
15	0.0566	0.98	0.054	0.88
Expt. 3				
5	NA	NA	0.0503	0.87
10	0.1277	0.99	0.0568	0.92
15	0.0895	0.99	0.0613	0.88

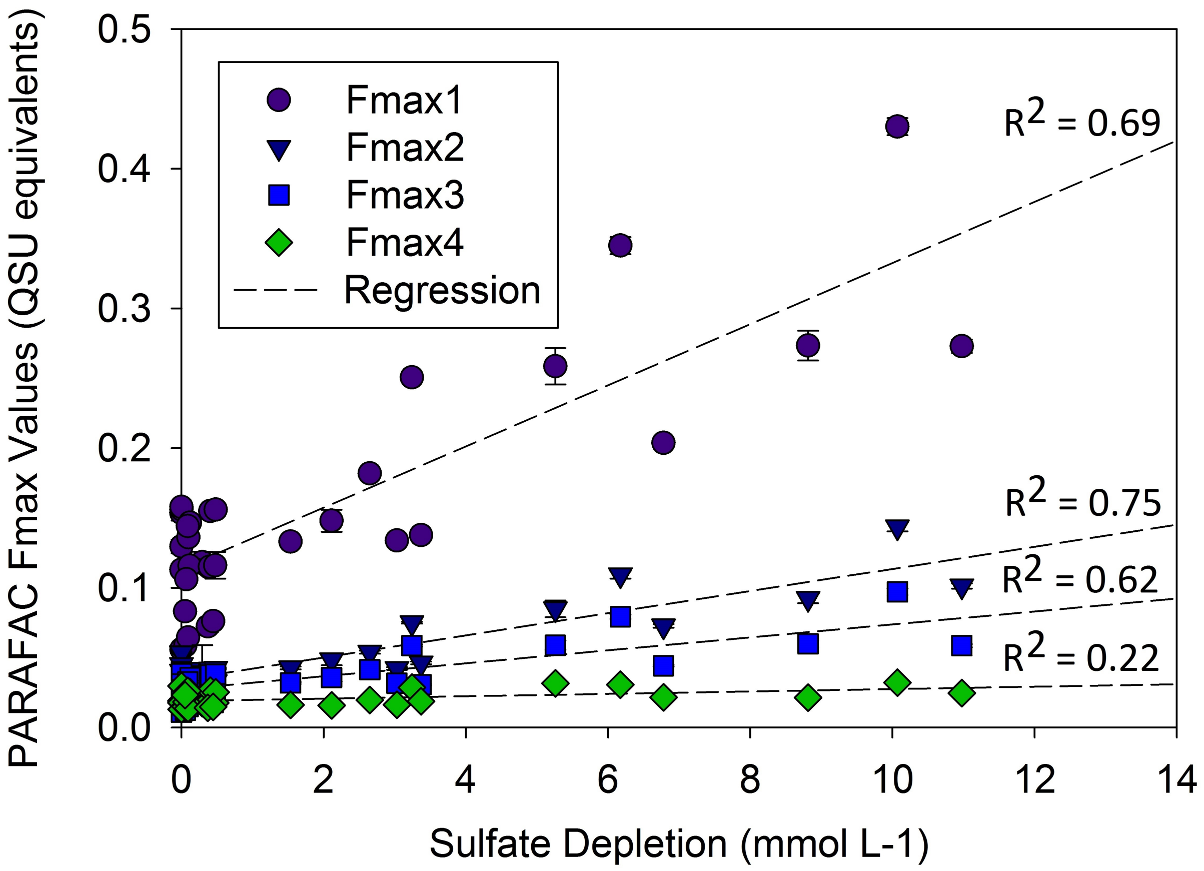

Fig. S6. Sulfate depletion versus the intensity of individual PARAFAC components in quinine sulfate equivalents (QSU) within each sample for experiment 2 with SRNOM addition. A linear regression for each component is shown. Standard error on slopes: Fmax1=0.02±0.0025, Fmax2=0.0079±0.00078 Fmax3=0.0046±0.00062, Fmax4= 0.00086±0.00027.

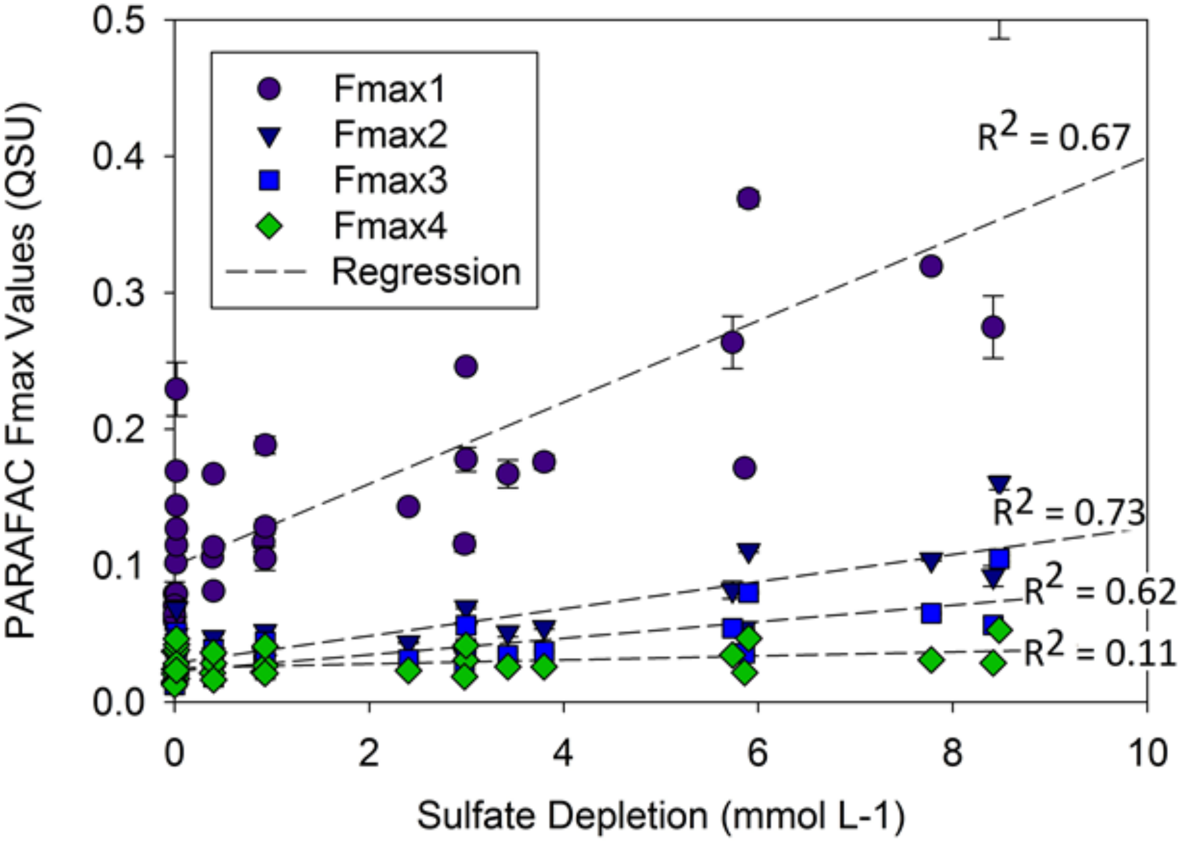

Fig. S7. Sulfate depletion versus the intensity of individual PARAFAC components in quinine sulfate equivalents (QSU) within each sample for experiment 3 with SRNOM and iron addition. A linear regression for each component is shown. Standard error on slopes: C1=0.03±0.0035, C2= 0.0099±0.001, C3=0.0061±0.0008, C4=0.0015±0.00063.

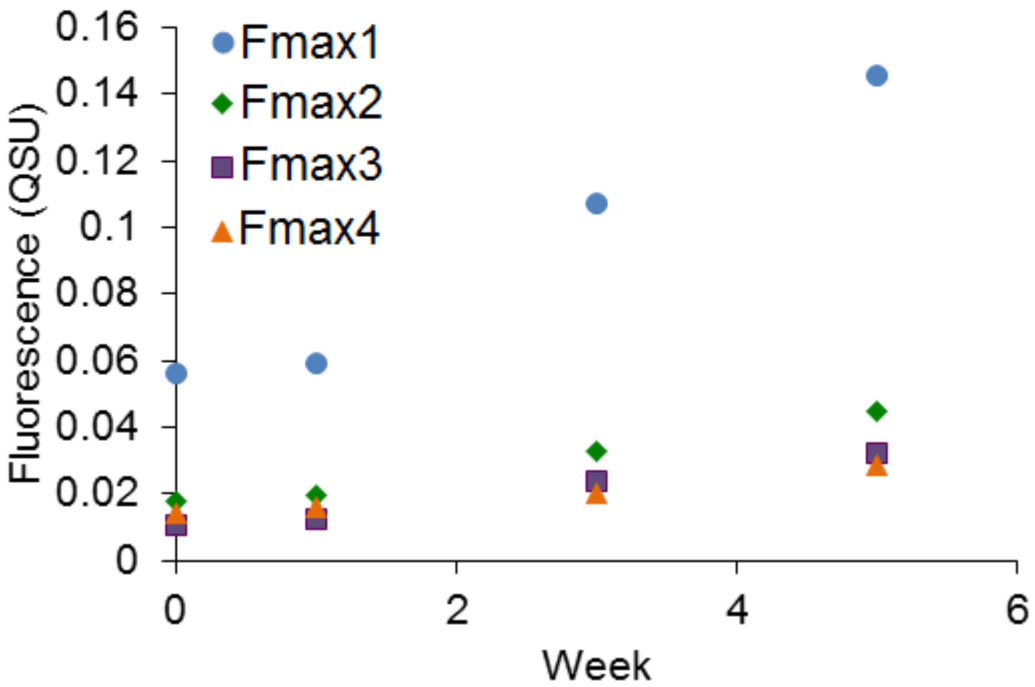

Figure S8. EEM-PARAFAC component changes during Experiment 1 during the fermentation (acetate only) treatment.

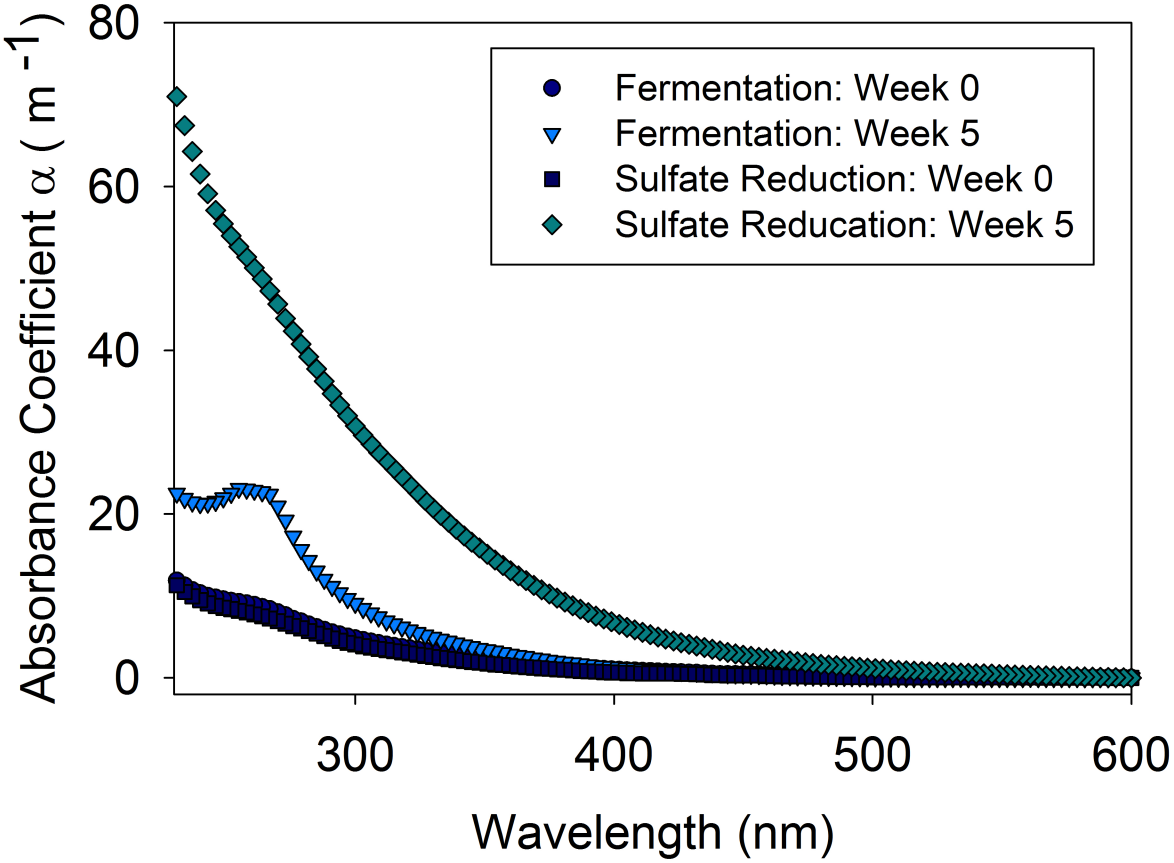

Fig. S9. Absorbance coefficient changes during experiment 1 sulfate reduction (initial sulfate concentration = salinity 15) and fermentation only treatment (acetate only).
